# Effects of Fermented Navel Orange Pulp on Growth Performance, Carcass Characteristics, Meat Quality, Meat Nutritional Value, and Serum Biochemical Indicators of Finishing Tibetan Pigs

**DOI:** 10.3390/foods13121910

**Published:** 2024-06-18

**Authors:** Chuanhui Xu, Pingwen Xiong, Wenjing Song, Qiongli Song, Yan Hu, Tongxing Song, Huayuan Ji, Xiaolian Chen, Zhiheng Zou

**Affiliations:** 1 Institute of Animal Husbandry and Veterinary Science, Jiangxi Academy of Agricultural Sciences, Nanchang 330200, China; xuchuanhui0620@163.com (C.X.); xiongpingwen1990@163.com (P.X.); 13397103983@163.com (W.S.); songqiongli1975@126.com (Q.S.); jhy478731@163.com (H.J.); 2 Jiangxi Provincial Key Laboratory of Green and Healthy Breeding of Livestock and Poultry, Nanchang 330200, China; 3 Institute of Animal Science and Fisheries, Gannan Academy of Sciences, Ganzhou 341401, China; 13517977277@163.com; 4 College of Animal Science and Technology, Huazhong Agricultural University, Wuhan 430070, China; songtongxing@mail.hzau.edu.cn

**Keywords:** fermented navel orange pulp, growth performance, carcass characteristics, meat quality, meat nutritional value, serum biochemical indicators, finishing Tibetan pigs

## Abstract

In order to cope with the limited supply of feed for global animal production, there is a pressing need to explore alternative feed resources. Orange pulp, a by-product of agriculture and industry, has shown potential to positively or neutrally impact pig productive performance when included in their diet. However, there is a lack of research on the effects of fermented navel orange pulp (FNOP) on pig growth and productive performance. This study aimed to investigate the effects of FNOP as a dry matter substitute on pig’s growth performance, carcass characteristics, meat quality, meat nutritional value, and serum biochemical indicators. The experiment involved 128 finishing Tibetan pigs, divided into four feed treatment groups, with varying levels (0%, 5%, 10% and 15%) of FNOP replacing dry matter in the basal diet. The results indicate that substituting 5% to 15% FNOP had no adverse effects on pig growth performance. However, at a 15% substitution rate, there was a decrease in serum growth hormone and IGF-1 levels, along with an increase in the feed-to-gain ratio. A 10% FNOP replacement notably increased the loin-eye muscle area of pigs. Additionally, 5% and 10% FNOP substitutions reduced the drip loss of pork. The study also found that substituting 5% to 15% FNOP increased unsaturated fatty acids and umami nucleotide contents in pork and raised serum total protein and uric acid (nucleotide-metabolism-related product) levels. These findings suggest that moderate FNOP substitution might improve meat quality, nutritional value, and maintain growth and productive performance in Tibetan pigs by improving protein synthesis and nucleotide metabolism, while also reducing feed costs. The optimal substitution ratio identified was 10%.

## 1. Introduction

The global supply of feedstock for animal production is a pressing issue. The increasing demand for animal products worldwide is met with limited feed-crop availability due to climate change and competition with human food sources [1]. Farmers also face rising production costs and challenges due to industrial competition and limited land resources [2]. To address this, exploring unconventional feed resources is crucial. Utilizing agricultural and industrial by-products not only promotes circularity in the food system but also reduces the environmental impact of pork production by recycling non-edible plant materials. According to the Food and Agriculture Organization of the United Nations, there are at least 1.6 billion tons of agricultural by-products globally each year, and their utilization can significantly reduce carbon dioxide emissions, lessening the negative environmental impacts and economic losses [3,4].

Citrus fruits are a major global fruit, with annual production exceeding 140 million tons [5]. Approximately 30% of citrus fruits, mainly oranges, are processed into juice, resulting in a substantial amount of citrus pulp, which can make up 49–69% of the processed fresh fruit’s weight [6]. Several studies have reviewed the chemical composition of citrus pulp [7,8], showing its nutritional value is comparable to conventional animal feed and holds promise for the animal feed industry [9,10]. Dehydrated citrus pulp has been successfully used in various livestock and ruminant feeds, including those for pigs [11,12,13]. However, almost two-thirds of fresh citrus pulp is discarded due to its high moisture content and perishability [14], with only a small portion being utilized by producers near processing facilities. In intensive pig farming, the high-moisture citrus pulp poses challenges for mechanized feed production, leading to additional energy costs for drying and pelleting.

In recent years, the popularity of liquid feed feeding models and advancements in biological fermentation technology have significantly broadened the potential applications of fresh citrus pulp in pig farming. Microbial fermentation not only prolongs the shelf life of high-moisture feed ingredients but also effectively breaks down anti-nutritional factors and promotes the production of probiotics and their beneficial metabolites [15,16]. Research indicates that incorporating fermented feed into diets can improve animal growth performance and meat quality [17,18,19,20]. Despite this, there is currently limited research on the utilization of fermented citrus pulp in pig feed.

The nutritional value of citrus pulp is influenced by various factors, such as the type of fruit, variety, season, ripeness, juicing method, and post-processing treatments like dehydration, ensilage, and fermentation [7]. This study specifically examines fresh by-products, focusing on navel orange pulp from Gannan navel oranges, a local citrus resource in China. It investigates the effects of fermented navel orange pulp (FNOP) in the diet of Tibetan pigs, a native breed in China. The research evaluates how substituting FNOP for different proportions of the basal diet dry matter (DM) impacts the growth performance, carcass characteristics, meat quality, meat nutritional value, and blood biochemical indicators of finishing Tibetan pigs. The results suggest that moderate FNOP usage as a substitute in feed does not negatively affect the growth performance or carcass characteristics of Tibetan pigs. Furthermore, it can improve the meat quality and nutritional value of the pork, showing promise in enhancing pork quality and reducing feed expenses.

## 2. Materials and Methods

### 2.1. Animal Ethics Statement

All procedures were approved by the Institutional Animal Care and Use Committee at Jiangxi Academy of Agricultural Sciences (Ethical Committee Number: 2024-JXAAS-XM-15).

### 2.2. Preparation of Fermented Navel Orange Pulp

Fresh navel orange pulp, a by-product obtained after juicing “Gannan navel oranges” harvested in November, was sourced from Dingcheng Agricultural Products Firm in Xinfeng County, Ganzhou City, Jiangxi Province, China. The navel orange pulp was crushed into small 1–2 cm pieces using a grinder. A mixture containing 97.3% navel orange residue, 2% rice mill by-product, and 0.7% sodium nitrate was blended thoroughly in a horizontal mixer. Additionally, a fermentation agent consisting of *Lactobacillus*, *Bacillus subtilis*, and yeast (sourced from the Feed Research Institute of the Chinese Academy of Agricultural Sciences in Beijing, with a live bacteria count of at least 105 CFU/g) was sprayed at a rate of 50 g per ton of weight during mixing. Prior to application, the fermentation agent was dissolved in livestock drinking water at a 1:1 ratio and allowed to activate for 1–2 h. The resulting mixture was then packed into 50 kg polyethylene bags, each fitted with a one-way valve to facilitate gas release without contamination. These bags were placed in a controlled environment at 20–30 °C for 7 days to undergo fermentation before being opened and utilized.

Chemical analysis of FNOP was carried out following the AOAC International guidelines (2005), and the results can be found in Table 1.

### 2.3. Experimental Design and Diets

The animal feeding experiment was conducted at a commercial breeding farm, Ganzhou Youdao Agricultural Development Co., Ltd., located in Ganzhou City, Jiangxi Province, China. The farm is equipped with comprehensive environmental control facilities and follows standardized management practices. A total of 128 finishing Tibetan pigs (body weight = 30.78 ± 1.04 kg) were randomly assigned to 4 dietary treatments with 4 replications each (8 pigs per replication). The dietary treatments consisted of the basal diet (CON) and experimental diets where FNOP replaced 5% (5% FNOP), 10% (10% FNOP), and 15% (15% FNOP) of the basal diet DM, respectively. The basal diet formulation comprehensively referred to the Chinese National Feed Standard for swine (lean-fat-type pig) and the recommended nutritional requirements for finishing Tibetan pigs [22,23], as outlined in Table 2. Pigs were weighed after 3 days of pre-feeding and the experiment lasted 49 days. All pigs had ad libitum access to water, while feed intake was controlled. Initial feed intake for experimental pigs was calculated by multiplying the average body weight by a coefficient of 0.04, with subsequent weekly increases of 10% based on the previous week’s intake. Pigs were fed 3 times daily at 07:30, 13:30, and 17:30.

### 2.4. Growth Performance Measurements

Throughout the experiment, the pigs’ feed intake was meticulously recorded on a per replicate basis, allowing for the calculation of the average daily feed intake (ADFI) per pig. On the 49th day of the experiment, the pigs were weighed after an overnight fast, and the average body weight, average daily gain (ADG), and feed conversion ratio (F:G) of each pig were calculated.

### 2.5. Sample Collection and Carcass Characteristics Measurements

On the 49th day of the experiment, a pig was selected from each replicate based on their closeness to the average weight of replicates and their moderate physical condition, as assessed visually. Following a 12 h fast, the live weight was measured; subsequently, the pigs were stunned via electrocution and slaughtered. Throughout the slaughter process, the hot carcass weight of each pig was measured on-site to determine the dressing percentage. Additionally, a tape measure was utilized to record the straight length and chest circumference. The backfat thickness at the thickest part of the shoulder, thoracolumbar junction, and lumbosacral junction were recorded and used to calculate the average backfat value. In accordance with the *Rules for Performance Testing of Breeding Pig in China*, a caliper was used to measure the maximum length and height of the cross-section of the *longissimus dorsi* muscle (LDM) at the 6th and 7th ribs of the thoracic vertebrae. The formula for calculating the loin-eye muscle area is as follows [25]:

$$\mathrm{Loin}-\mathrm{eye}\;\mathrm{muscle}\;\mathrm{area}\;({\mathrm{cm}}^{2})=Length\left(cm\right)\times Width\left(cm\right)\times 0.7$$

### 2.6. Meat Quality Measurements

The LDM samples stored at 4 °C were used to analyze the pH value, meat color, drip loss, shear force, and marbling score. The assessment of meat quality followed the methodology outlined in a previous study [26]. After slaughter, the pH of each LDM sample was measured at 45 min and 24 h using a portable pH meter (pH-Star, Matthäaus GmbH, Päottmes, Germany). Meat color values, including lightness (L*), redness (a*), and yellowness (b*), were assessed at the same time points post-slaughter using a colorimeter (CR-10, Konica Minolta, Osaka, Japan). The color difference (ΔE*) between the two time points was calculated using a specific formula, as follows:

$$\Delta E*=\sqrt{{\left(\Delta L*\right)}^{2}+{\left(\Delta a*\right)}^{2}+{\left(\Delta b*\right)}^{2}}.$$

Approximately 10 g of each LDM sample was placed in a sealed plastic tube at 4 °C, and after 24 h, the surface moisture was removed before weighing to determine drip loss. The shear force was measured using a C-LM4 tenderness tester (Tenovo International Co., Limited, Beijing, China), following the manufacturer‘s instructions. The marbling score was determined 45 min post-slaughter based on the NPPC meat color chart (Nanjing Mingao Instrument Equipment Co., Ltd., Nanjing, China).

### 2.7. Meat Conventional Nutrition

The contents of moisture, crude protein (CP), ether extract (EE), cholesterol, inosine monophosphate (IMP), and adenosine monophosphate (AMP) of each LDM sample were determined using Association of Official Analytical Chemists (AOAC) methods (2005). The samples were cut into pieces, ground into a paste using a high-speed universal crusher (FW100, Taisite Ltd., Tianjin, China), and placed into sample cups. Moisture content (%) was calculated by measuring weight loss after oven-drying the samples (3 g) at 102 °C for 12 h (until constant weight) in a Memmert laboratory dryer (UN 75, Schwabach, Germany). Crude protein content (%) was determined using the Kjeldahl method with an automatic Kjeldahl nitrogen analyzer (SKD-200, Shanghai Peiou Analysis Instruments Co., Ltd., Shanghai, China). Fat content (%) was measured using the Soxhlet method with petroleum ether extraction in a Hanon Automatic Soxhlet Extractor (SZF-06A, Shanghai Lichen Instruments Technology Co., Ltd., Shanghai, China). The levels of IMP, AMP, and cholesterol were determined using a high-performance liquid chromatography system (Agilent 1200, Agilent Technologies, Santa Clara, CA, USA).

### 2.8. Amino Acid Composition

Approximately 0.1 g of each LDM sample was weighed and digested with 5 mL of 6 mol/L HCl solution at 105 °C in an oven for 24 h. The volume was then adjusted to 50 mL in a volumetric flask, and the sample was filtered through a 0.22 mm water phase filter into a centrifuge tube. Subsequently, 2 mL of the filtrate was evaporated in an evaporating dish in a 60 °C water bath, followed by the addition of 4 mL of 0.02 mol/L HCl solution. Once dissolved, the sample was stored at 4 °C for detection using an ion-exchange AA analyzer (L8900, Hitachi, Tokyo, Japan).

### 2.9. Fatty Acid Profile

The fatty acid profile was analyzed using gas chromatography (GC), as outlined in the study by Hao et al., 2020 [27]. The LDM samples were first extracted using a mixture of chloroform and methanol (2:1; *v*/*v*). Approximately 20 g of each LDM sample was weighed and dried at 105 °C for 1 h, followed by weighing 1 g of the dried sample and leaching it with petroleum ether for 3 h. Subsequently, 60 mg of the extracted fat was dissolved in 4 mL of isooctane, with the addition of 200 mL of potassium hydroxide-methanol and 1 g of sodium bisulfate. After salt precipitation, the solution containing the methyl esters was separated into the upper layer and stored in a refrigerator at 4 °C. Prior to GC detection (Model 7890 A, Agilent Technologies, CA, USA), each sample was filtered through a 0.22 nm filter membrane. The fatty acid concentration was then determined using GC ChemStation software B.04.03 (Agilent Technologies, CA, USA).

### 2.10. Serum Sample Collection and Serum Biochemical Measurements

On the 49th day, another three pigs were randomly selected from each replicate. Fasting blood samples (5 to 10 mL) were collected from the anterior vena cava after a 12 h fast. The blood was collected into sterile EP tubes without any anticoagulant. After centrifugation at 3000 rpm for 5 min, the supernatant was stored at −80 °C for future use. Serum total protein (TP), blood urea nitrogen (BUN), and triglyceride levels were analyzed using a fully automatic biochemical analyzer (PBC22A Plus, LWPOCT, Shenzhen, China). Serum uric acid (UA) levels were determined using a colorimetric assay kit (Nanjing Jiancheng Bioengineering Institute, Nanjing, China). Growth hormone (GH) and insulin-like growth factor-1 (IGF-1) levels were measured using an enzyme-linked immunoassay kit (Nanjing Jiancheng Bioengineering Institute, Nanjing, China).

### 2.11. Statistical Analyses

All data in the current study were analyzed via one-way analysis of variance (ANOVA) using the statistical software SPSS 20.0 (Chicago, IL, USA), where the type of diet was considered as the main effect, followed by Duncan’s multiple range analysis. Statistically significant differences were defined as *p* < 0.05, highly significant statistical differences as *p* < 0.01, and a trend towards significance as *p* < 0.10. All results are shown as the means ± standard deviation.

## 3. Results

### 3.1. Growth Performance

In this study, we investigated the effects of substituting FNOP for DM in the base diet on the growth performance of finishing Tibetan pigs by standardizing feed intake. The results presented in Table 3 show that there were no significant differences in ADFI across the experimental groups, indicating consistent control over feed consumption. Additionally, the ADG did not vary significantly among the experimental groups. It is noteworthy that the feed-to-gain ratio in the 15% FNOP group increased by 0.26 compared to the CON group, although this difference was not statistically significant. These findings suggest that replacing 5% to 15% of the base diet DM with FNOP during the finishing phase does not have a negative impact on the growth performance of Tibetan pigs. Moreover, a 15% replacement ratio may reduce the feed conversion rate.

### 3.2. Carcass Characteristics

The impact of FNOP on the carcass characteristics of finishing Tibetan pigs was further explored, with detailed results presented in Table 4. The live weight did not differ significantly among the three FNOP groups compared to the CON group, although the 15% FNOP group exhibited a lower live weight than the 5% and 10% FNOP groups (*p* < 0.05). In contrast to the CON group, the loin-eye muscle area significantly increased in the 10% FNOP group (*p* < 0.05). No other carcass characteristic indicators showed significant differences across the experimental groups. These results suggest that substituting 5% to 15% of the base diet with FNOP does not negatively impact the productive performance of Tibetan pigs, and a 10% replacement may even improve lean meat production.

### 3.3. Meat Quality

Table 5 illustrates the impact of FNOP on the meat quality of finishing Tibetan pigs. In comparison to the CON group, the 10% FNOP group displayed a tendency towards decreased pH_24h_ values in the meat (*p* < 0.10). The 5% FNOP group exhibited the highest L*_45min_ value (*p* < 0.05). Moreover, the 15% FNOP group demonstrated the most significant color difference (ΔE*_(45min–24h)_) (*p* < 0.01), correlating with the maximum pH fluctuation during the meat’s acidification phase. Additionally, both the 5% and 10% FNOP groups experienced lower drip loss in the meat when compared to the CON group and the 15% FNOP group (*p* < 0.05). These findings indicate that substituting 5% and 10% FNOP improves meat quality, with the 10% replacement ratio showing superior acidification effects.

### 3.4. Meat Nutritional Value

The study further examined the nutritional composition of the meat and found no significant differences in moisture, CP, EE, ash, and cholesterol content among the meat from all experimental groups (Table 6). However, there was a notable increase in the content of IMP in the meat from the 5%, 10%, and 15% FNOP groups by 103.28%, 123.78%, and 85.97%, respectively, compared to the CON group (*p* < 0.01) (Table 7). Additionally, the content of AMP in the meat from the 5% and 10% FNOP groups showed a significant increase of 20.18% and 18.30%, respectively (*p* < 0.01) (Table 6). The amino acid composition did not vary significantly across the experimental groups (Table 7). The fatty acid profile of pork is depicted in Table 8. The levels of C18:0 and SFA in the 5%, 10%, and 15% FNOP groups exhibited a significant linear decrease compared to the CON group (*p* < 0.01), while the levels of C18:1n9 and MUFA increased significantly (*p* < 0.05 and *p* < 0.01, respectively). Moreover, the levels of C18:2n6 and PUFA significantly increased in the 10% and 15% FNOP groups (*p* < 0.01 and *p* < 0.05, respectively). The UFA:SFA ratio in the meat from the 5%, 10%, and 15% FNOP groups displayed a linear, significant increase compared to the CON group (*p* < 0.01). These results indicate that FNOP substitution leads to higher levels of unsaturated fatty acids and umami nucleotides in the meat, with the most pronounced effects observed at the 10% replacement level.

### 3.5. Serum Biochemical Indicators

Table 9 displays the serum biochemical indicators related to growth and metabolism. The serum TP levels in all FNOP experimental groups were significantly higher compared to the CON group (*p* < 0.01), while there was no significant difference in the levels of BUN, a product of protein metabolism. This suggests a potential promotion of protein synthesis metabolism in pigs. Moreover, the levels of UA, a purine metabolism product, increased notably in the 5%, 10%, and 15% FNOP groups compared to the CON group (*p* < 0.01). The 15% FNOP group also exhibited a significant rise in serum triglyceride levels compared to the CON group (*p* < 0.05). In terms of growth-related hormones, there was a linear and significant decrease in GH levels with increasing FNOP replacement (*p* < 0.01). Additionally, IGF-1 levels were notably reduced in the 15% FNOP group compared to the CON group (*p* < 0.05). These results suggest that FNOP replacement enhances protein synthesis metabolism and nucleotide metabolism in pigs, but exceeding 10% may lead to a reduction in growth-related hormone levels.

## 4. Discussion

This study aimed to investigate the effects of replacing 5%, 10%, and 15% of the basal diet DM with FNOP on the growth performance, carcass characteristics, meat quality, nutritional value of meat, and blood biochemical indicators in finishing Tibetan pigs. Our findings demonstrated that moderate replacement with FNOP did not negatively impact pig growth or productive performance. Moreover, FNOP also enhanced the metabolic processes related to nucleotides and protein synthesis, which ultimately improved the meat quality and nutritional value of the pork.

FNOP in this study was characterized by higher protein and neutral detergent fiber contents than unfermented citrus pulp, as reported in previous studies [8,13], while showing lower levels of carbohydrates and energy. These observations suggest that biofermentation significantly modified the chemical composition of the navel orange pulp. The high level of neutral detergent fiber content can be attributed to the rich content of hemicellulose, which may increase the fermentability of FNOP fiber in the gastrointestinal tract [28,29,30].

Previous studies have indicated that the addition of 15% citrus pulp to the diet of finishing pigs is appropriate [31,32]. In the present study, the replacement of 5%, 10%, and 15% of the basal diet DM with FNOP led to a slight decrease in the energy and protein content of the diet. However, under the condition of uniform feed intake, the growth performance of the pigs was not significantly adversely affected. However, it is important to note that when the replacement ratio reached 15%, there was a decrease in the feed conversion efficiency of the pigs, potentially linked to the reduced energy and protein density of the diet. Furthermore, at the 15% FNOP substitution level, a notable reduction in serum growth-related hormone levels was detected, which could be associated with the decrease in their average daily DE intake (4.56 Mcal/d compared to the CON group’s 4.67 Mcal/d).

Regarding carcass characteristics, the live weight of pigs at slaughter with a 15% FNOP substitution ratio was lower compared to those with 5% and 10% ratios, which may be related to the random principle followed in the selection process. In this trial, FNOP substitution did not significantly affect most carcass characteristics, including carcass weight, yield, body length, chest circumference, and backfat thickness, contrasting with Ferrer et al.’s findings. They noted a linear decrease in carcass weight and backfat gain as the proportion of dehydrated citrus pulp in the diet increased [13]. These discrepancies may be attributed to differences in pig breeds and the citrus pulp materials used.

When FNOP replaced 10% of the basal diet DM, there was a significant increase in the loin-eye muscle area of the pigs’ LDM, indicating a positive effect of FNOP on promoting protein deposition in pigs. This is further supported by the increased total protein (TP) levels in blood biochemical indicators. Bakare et al. observed a similar trend, with the serum TP level of finishing pigs initially increased and then decreased with the addition of dehydrated orange pulp in the diet, peaking at a 24% addition level [32]. This aligns with the results of the current study. In the study by Bakare et al., it was observed that as the level of dehydrated citrus pulp added to the diet increased, the protein content decreased. However, at a 24% addition ratio, the crude protein level can reach 14.76%, which still exceeds the NRC (2012) recommendations for meeting the protein deposition needs of finishing pigs. This may be an important factor in increasing their serum TP levels. Belloumi et al. found that incorporating dehydrated citrus pulp into pig diets could potentially enhance nitrogen absorption and utilization in the intestines [33], evidenced by a decrease in fecal metabolites associated with bacterial protein fermentation and an increase in serum metabolites related to protein metabolism. Cui et al. suggested that citrus extracts may also boost nitrogen absorption and utilization in pigs by improving intestinal morphology and digestive enzyme activity [34]. Additionally, Noh et al. showed that a diet supplemented with a product fermented using *Bacillus subtilis* on a mixture of citrus pulp and fish by-product improved the digestibility of various nutrients in pigs [35]. The observed increase in loin-eye muscle area and TP levels in our current experiment could be a result of enhanced nitrogen digestion and absorption in the pigs’ intestines. However, it is important to note that a 15% FNOP substitution did not lead to an improvement in loin-eye muscle area, possibly due to reduced levels of GH and IGF-1.

Meat quality traits are intricate quantitative features comprising various indicators like meat color, pH value, water-holding capacity, shear force, and marbling. Following slaughter, pork typically undergoes an aging process lasting 24 to 36 h, during which rigor mortis occurs and resolves. A key biochemical change during this period is the conversion of glycogen to lactate through anaerobic glycolysis, resulting in a decrease in pH value. In our study, we observed that pork from the 5% and 10% FNOP groups exhibited appropriate pH_24h_ values, while the pH_24h_ values of the CON group and the 15% FNOP group remained above 6. Postmortem aging contributes to enhancing the meat’s water-holding capacity, thereby improving the juiciness of pork [36], as evidenced by reduced drip loss in the 5% and 10% FNOP groups. A lower final pH value generally indicates a higher glycogen content in the muscle at slaughter, which is linked to the muscle’s activity level and metabolic state, although this aspect was not further explored in our study. Concurrent with pH changes, alterations in meat color occur; notably, the 10% FNOP group showed the greatest color difference from 45 min to 24 h after slaughter. Throughout the aging process, the lightness value (L*) and yellowness value (b*) of pork from all experimental groups increased, consistent with previous research findings [37,38]. Particularly, the 24 h lightness value of pork from the 10% FNOP group significantly improved, possibly due to enhanced protein denaturation and structural changes resulting from the pH decrease. The denaturation of sarcoplasmic proteins and increased transverse spacing of myofibrils contribute to heightened light scattering [39,40].

The nutritional quality and taste of meat play a crucial role in how consumers perceive it. The consumption of fatty acids is intricately linked to consumer health. Research suggests that consuming excessive saturated fatty acids may elevate the risk of developing type 2 diabetes and heart disease [41], whereas unsaturated fatty acids have shown to have positive effects on health, such as reducing inflammation, regulating glycolipid metabolism, and supporting muscle growth. In this study, replacing 5%, 10%, and 15% of the basal diet DM with FNOP led to an increase in monounsaturated fatty acids and a decrease in saturated fatty acids in pork. Notably, substituting 10% of the basal diet DM with FNOP significantly raised the levels of polyunsaturated fatty acids. Ferrer et al. (2022) discovered that supplementing dehydrated orange pulp into the diet led to an increase in total fatty acid content and the MUFA/SFA ratio, resulting in decreased SFA content, increased MUFA content, and an increased MUFA/SFA ratio in the LDM of pigs [13], consistent with the findings of this study. Moreover, Liu et al. (2023) observed that fermenting a mixture of corn, soybean meal, and wheat bran with *Enterobacter faecium* and *Bacillus subtilis* decreased the SFA level and increased MUFA and PUFA levels in the mixture. The inclusion of this fermented mixture in the diet upregulated the expression of *ACAA1* and *FADS2* genes related to unsaturated fatty acid synthesis in muscle tissue, leading to an increase in PUFA content and the PUFA/SFA ratio in pork [18]. The concurrent changes in the muscle fatty acid profile and dietary fatty acid profile in these studies support the notion that the inclusion of orange pulp and pre-fermentation impact the dietary fatty acid profile, potentially influencing the fatty acid profile of pork observed in this experiment.

Nucleotides play a crucial role in the nutritional and flavor profile of pork products, particularly IMP, which enhances the umami taste of meat [42]. This study observed that substituting 5%, 10%, and 15% FNOP increased IMP levels in pork, with 5% and 10% FNOP also boosting AMP levels. These findings align with prior research indicating that fermented feed can upregulate genes linked to IMP synthesis in muscle tissue, thereby enhancing IMP concentration [19]. This phenomenon is likely attributed to the heightened nucleotide production facilitated by microbial fermentation processes [43,44]. The higher serum UA levels across all FNOP groups, as a by-product of purine metabolism, indirectly suggest that fermented feed may stimulate nucleotide synthesis and accumulation.

## 5. Conclusions

This study found that substituting 5% to 15% of the basal diet DM with FNOP did not negatively impact the growth and productive performance of finishing Tibetan pigs. However, the substitution of 15% FNOP led to a decrease in the levels of growth-related hormones in the pigs’ serum and also resulted in a reduced feed conversion efficiency, suggesting that 15% may be the maximum threshold for the FNOP substitution ratio. The most optimal substitution ratio of FNOP appears to be 10%, as it maximizes the meat quality and nutritional value. This is supported by an increase in the loin-eye muscle area of the pigs’ LDM, a rise in unsaturated fatty acids and savory nucleotides in the pork, and a decrease in drip loss. While further research is needed to understand the specific mechanisms behind FNOP’s enhancement of pork quality in finishing pigs, this study highlights the potential benefits of FNOP in improving pork quality and reducing feed costs.

## Figures and Tables

**Table 1:** Chemical composition of fermented navel orange pulp (as 88% DM basis).

Item	FNOP
Digestible energy, Mcal/kg ^1^	1.91
Crude protein, % ^2^	11.09
Ether extract, % ^2^	3.03
Carbohydrates, % ^2^	19.07
Ash, % ^2^	5.26
Crude fiber, % ^2^	49.55
Neutral detergent fiber, % ^2^	35.34
Acid detergent fiber, % ^2^	11.83

Note: ^1^ Calculated value, digestible energy = [949 + (0.789 × GE) − (43 × %ash) − (41 × %NDF)]/1000 [21]. ^2^ Analyzed values. FNOP = fermented navel orange pulp.

**Table 2:** Ingredients and nutrient levels of experimental diets (%, as-fed basis).

Item	CON	5% FNOP	10% FNOP	15% FNOP
Ingredient, %				
Corn	35.55	33.77	32.00	30.22
Soybean meal	6.20	5.89	5.58	5.27
Rice bran meal	45.00	42.75	40.50	38.25
Wheat bran	8.34	7.92	7.51	7.09
FNOP	0.00	5.00	10.00	15.00
Calcium hydrogen phosphate	0.87	0.83	0.78	0.74
Calcium carbonate	1.09	1.04	0.98	0.93
Salt	0.35	0.33	0.32	0.30
Baking soda	0.20	0.19	0.18	0.17
L-lysine sulfate (70%)	0.15	0.14	0.14	0.13
L-threonine (98.5%)	0.15	0.14	0.14	0.13
Choline chloride	0.05	0.05	0.05	0.04
Bentonite	1.00	0.95	0.90	0.85
Mildewcide ^1^	0.05	0.05	0.05	0.04
Premix ^2^	1.00	0.95	0.90	0.85
Calculated composition ^3^				
DE, Mcal/kg	2.70	2.66	2.62	2.58
CP, %	13.69	13.56	13.43	13.30
NDF, %	19.73	21.47	23.21	24.95
ADF, %	7.83	8.34	8.85	9.35
SID Lys, %	0.53	0.52	0.52	0.51
Ca, %	0.67	0.68	0.69	0.70
STTD phosphorus, %	0.38	0.37	0.35	0.34
Analyzed composition				
CP, %	13.58	13.28	13.21	13.18

Note: ^1^ Mildewcide, ammonium propionate; ^2^ Supplied per kilogram of the diet: 60 mg Fe, 8 mg Cu, 20 mg Mn, 80 mg Zn, 0.35 mg I, 0.2 mg Se, 5250 IU vitamin A, 1125 IU vitamin D3, 75 mg vitamin E, 2.25 mg vitamin B1, 7.5 mg vitamin B2, 3 mg vitamin B6, 0.03 mg vitamin B12, 24 mg pantothenic acid, 0.9 mg folic acid, 30 mg niacin, 0.15 mg biotin, and 12 mg antioxidant. ^3^ Calculated values based on *Tables of Feed Composition and Nutritional Values in China* (2022) [24]. CON = basal diet; FNOP = fermented navel orange pulp; DE = digestible energy; CP = crude protein; NDF = neutral detergent fiber; ADF = acid detergent fiber; SID Lys = standardized ileal digestible lysine; STTD phosphorus = standardized total tract digestibility of phosphorus.

**Table 3:** Effects of fermented navel orange pulp on the growth performance of finishing Tibetan pigs.

Item	CON	5% FNOP	10% FNOP	15% FNOP	*p*-Value
ADFI, kg/d	1.73 ± 0.33	1.75 ± 0.33	1.77 ± 0.34	1.77 ± 0.31	0.635
Initial weight, kg	31.12 ± 0.58	30.39 ± 1.16	30.94 ± 1.03	30.67 ± 1.49	0.802
Final weight, kg	46.76 ± 0.84	46.10 ± 1.61	46.91 ± 1.22	45.89 ± 1.14	0.602
ADG, g/d	319.26 ± 10.52	320.63 ± 10.21	325.83 ± 13.39	310.75 ± 11.33	0.352
F:G	5.44 ± 0.18	5.47 ± 0.18	5.45 ± 0.20	5.70 ± 0.23	0.251

Note: Data are expressed as means ± standard deviation, *n* = 32. CON = basal diet; FNOP = fermented navel orange pulp; ADFI = average daily feed intake; ADG = average daily gain; F:G = feed-to-gain ratio.

**Table 4:** Effects of fermented navel orange pulp on the carcass traits of finishing Tibetan pigs.

Item	CON	5% FNOP	10% FNOP	15% FNOP	*p*-Value
Live weight, kg	47.76 ± 5.01 ^ab^	50.54 ± 3.29 ^b^	49.79 ± 0.38 ^b^	43.16 ± 1.51 ^a^	0.023
Carcass weight, kg	30.41 ± 4.14	34.15 ± 2.77	32.10 ± 1.03	29.34 ± 1.97	0.121
Carcass yield, %	63.51 ± 2.42	67.57 ± 3.07	64.46 ± 1.72	67.97 ± 4.00	0.127
Body length, cm	77.00 ± 3.56	77.00 ± 7.70	75.50 ± 3.7	75.75 ± 4.99	0.963
Chest circumference, cm	83.00 ± 3.74	83.63 ± 7.65	83.70 ± 1.01	80.75 ± 1.71	0.756
BFT at thickest part of the shoulder, mm	36.24 ± 3.13	33.44 ± 5.52	34.38 ± 6.42	30.9 ± 3.41	0.688
BFT at thoracolumbar junction, mm	15.32 ± 1.73	15.99 ± 3.36	18.40 ± 6.79	14.64 ± 4.95	0.886
BFT at lumbar sacral junction, mm	20.86 ± 0.59	22.41 ± 5.79	22.73 ± 2.41	19.85 ± 3.70	0.864
BFT, mm	24.14 ± 1.49	23.94 ± 4.29	25.17 ± 4.34	21.80 ± 3.19	0.910
Loin-eye muscle area, cm^2^	8.42 ± 0.72 ^a^	9.20 ± 1.16 ^ab^	12.23 ± 1.71 ^b^	8.94 ± 1.17 ^ab^	0.017

Note: Data are expressed as means ± standard deviation, *n* = 4. CON = basal diet; FNOP = fermented navel orange pulp; BFT = backfat thickness. ^a,b^ Within a row, values with different superscripts differ significantly at *p* < 0.05.

**Table 5:** Effects of fermented navel orange pulp on the meat quality of finishing Tibetan pigs.

Item	CON	5% FNOP	10% FNOP	15% FNOP	*p*-Value
pH_45min_	6.74 ± 0.16	6.55 ± 0.16	6.53 ± 0.09	6.62 ± 0.18	0.274
pH_24h_	6.33 ± 0.21	5.82 ± 0.41	5.64 ± 0.24	6.14 ± 0.49	0.067
L*_45min_	34.95 ± 0.70	36.05 ± 0.52	35.06 ± 1.15	34.64 ± 1.44	0.286
a*_45min_	15.41 ± 0.71	16.76 ± 1.28	15.18 ± 0.47	16.25 ± 1.29	0.148
b*_45min_	0.38 ± 0.22 ^a^	1.16 ± 0.52 ^b^	0.30 ± 0.36 ^a^	0.41 ± 0.41 ^a^	0.032
L*_24h_	36.73 ± 3.29 ^a^	38.51 ± 1.08 ^a^	43.30 ± 2.94 ^b^	37.17 ± 2.7 ^a^	0.016
a*_24h_	15.40 ± 0.72	17.72 ± 1.11	16.62 ± 1.69	16.13 ± 0.78	0.079
b*_24h_	1.16 ± 0.53	1.45 ± 0.26	1.90 ± 0.17	1.30 ± 0.36	0.121
ΔE*_(45min–24h)_	2.51 ± 2.08 ^A^	2.98 ± 1.38 ^A^	8.70 ± 2.18 ^B^	2.98 ± 2.11 ^A^	0.002
Drip loss, %	1.38 ± 0.29 ^b^	0.86 ± 0.26 ^a^	0.98 ± 0.31 ^a^	1.26 ± 0.10 ^b^	0.010
Shearing force, kgf	5.95 ± 1.25	5.66 ± 1.07	5.73 ± 0.84	6.34 ± 0.76	0.777
Marbling score	2.88 ± 1.03	2.5 ± 0.41	2.5 ± 0.71	2.88 ± 0.75	0.806

Note: Data are expressed as means ± standard deviation, *n* = 4. CON = basal diet; FNOP = fermented navel orange pulp; ΔE*_(45min–24h)_ = color difference of meat from 45 min to 24 h. ^a,b^ or ^A,B^ Within a row, values with different superscripts differ significantly at *p* < 0.05 and *p* < 0.01, respectively.

**Table 6:** Effects of fermented navel orange pulp on the conventional nutrient levels of the longissimus dorsi muscle in finishing Tibetan pigs.

Item	CON	5% FNOP	10% FNOP	15% FNOP	*p*-Value
Moisture, %	70.72 ± 1.20	70.16 ± 0.88	70.95 ± 1.76	71.60 ± 0.26	0.405
CP, %	23.28 ± 0.93	23.65 ± 0.57	23.15 ± 0.64	23.62 ± 0.36	0.647
EE, %	1.26 ± 0.64	1.85 ± 1.23	1.52 ± 1.09	1.06 ± 0.60	0.670
Ash, %	1.23 ± 0.03	1.19 ± 0.05	1.31 ± 0.09	1.31 ± 0.09	0.086
IMP, g/kg	1.47 ± 0.22 ^A^	3.00 ± 0.07 ^BC^	3.29 ± 0.28 ^C^	2.73 ± 0.14 ^B^	0.004
AMP, mg/kg	73.05 ± 8.71 ^A^	87.79 ± 3.66 ^B^	86.42 ± 2.78 ^B^	69.99 ± 5.26 ^A^	0.001
Cho, g/kg	0.77 ± 0.08	0.73 ± 0.06	0.75 ± 0.02	0.79 ± 0.03	0.536

Note: Data are expressed as means ± standard deviation, *n* = 4. CON = basal diet; FNOP = fermented navel orange pulp; CP = crude protein; EE = ether extract; IMP = inosine monophosphate; AMP = adenosine monophosphate; Cho = cholesterol. ^A,B,C^ Within a row, values with different superscripts differ significantly at *p* < 0.01.

**Table 7:** Effects of fermented navel orange pulp on the free amino acid profile of the longissimus dorsi muscle in finishing Tibetan pigs (g/100 g, as-fresh basis).

Item	CON	5% FNOP	10% FNOP	15% FNOP	*p*-Value
EAA					
Lysine	2.35 ± 0.25	2.50 ± 0.20	2.54 ± 0.11	2.36 ± 0.14	0.387
Methionine	0.68 ± 0.06	0.72 ± 0.04	0.73 ± 0.03	0.68 ± 0.04	0.392
Valine	1.24 ± 0.12	1.32 ± 0.08	1.31 ± 0.06	1.23 ± 0.10	0.421
Isoleucine	1.07 ± 0.09	1.11 ± 0.05	1.12 ± 0.05	1.04 ± 0.07	0.336
Leucine	2.00 ± 0.19	2.11 ± 0.13	2.14 ± 0.06	1.97 ± 0.11	0.250
Phenylalanine	1.21 ± 0.11	1.26 ± 0.07	1.30 ± 0.05	1.19 ± 0.06	0.221
Histidine	1.14 ± 0.11	1.19 ± 0.05	1.18 ± 0.06	1.10 ± 0.06	0.255
Threonine	1.13 ± 0.11	1.20 ± 0.08	1.20 ± 0.05	1.10 ± 0.06	0.285
NEAA					
Aspartate	1.96 ± 0.17	2.06 ± 0.12	2.07 ± 0.09	1.94 ± 0.15	0.434
Serine	0.90 ± 0.08	0.93 ± 0.04	0.94 ± 0.03	0.88 ± 0.07	0.357
Glutamate	3.19 ± 0.29	3.31 ± 0.15	3.32 ± 0.15	3.08 ± 0.19	0.351
Glycine	0.99 ± 0.09	1.04 ± 0.05	1.03 ± 0.04	0.96 ± 0.05	0.306
Alanine	0.40 ± 0.54	0.43 ± 0.59	0.14 ± 0.01	0.12 ± 0.01	0.574
Cysteine	0.27 ± 0.02	0.28 ± 0.02	0.28 ± 0.01	0.26 ± 0.02	0.463
Tyrosine	1.07 ± 0.10	1.11 ± 0.04	1.11 ± 0.04	1.03 ± 0.07	0.268
Arginine	1.54 ± 0.13	1.59 ± 0.06	1.63 ± 0.06	1.52 ± 0.09	0.385
Proline	0.66 ± 0.06	0.68 ± 0.03	0.70 ± 0.03	0.64 ± 0.03	0.205
Total EAA	10.83 ± 1.04	11.40 ± 0.70	11.54 ± 0.46	10.67 ± 0.64	0.314
Total NEAA	10.99 ± 0.67	11.43 ± 0.38	11.21 ± 0.46	10.42 ± 0.66	0.118
Total AA	21.82 ± 1.61	22.83 ± 0.76	22.75 ± 0.92	21.09 ± 1.30	0.181
Umami amino acids ^1^	8.09 ± 0.47	8.42 ± 0.38	8.18 ± 0.35	7.62 ± 0.48	0.107

Note: Data are expressed as means ± standard deviation, *n* = 4. ^1^ Umami amino acids = Aspartate + Glutamate + Glycine + Alanine + Arginine. CON = basal diet; FNOP = fermented navel orange pulp; EAA = essential amino acid; NEAA = non-essential amino acid; AA = amino acid.

**Table 8:** Effects of fermented navel orange pulp on the fatty acid profile of the longissimus dorsi muscle in finishing Tibetan pigs (%).

Item	CON	5% FNOP	10% FNOP	15% FNOP	*p*-Value
C10:0	0.13 ± 0.01	0.12 ± 0.01	0.11 ± 0.01	0.12 ± 0.02	0.277
C12:0	0.05 ± 0.01 ^A^	0.09 ± 0.02 ^B^	0.06 ± 0.01 ^A^	0.05 ± 0.01 ^A^	0.009
C14:0	1.26 ± 0.05	1.25 ± 0.08	1.14 ± 0.08	1.21 ± 0.05	0.085
C16:0	26.15 ± 0.21	25.73 ± 0.40	26.05 ± 0.07	25.85 ± 0.19	0.244
C17:0	0.20 ± 0.02	0.17 ± 0.02	0.17 ± 0.02	0.18 ± 0.04	0.267
C18:0	12.97 ± 0.22 ^C^	12.40 ± 0.12 ^B^	11.78 ± 0.19 ^A^	11.76 ± 0.27 ^A^	<0.001
C20:0	0.42 ± 0.03	0.37 ± 0.03	0.40 ± 0.05	0.44 ± 0.02	0.053
C16:1	5.78 ± 0.12	6.08 ± 0.11	5.91 ± 0.38	5.95 ± 0.12	0.285
C17:1	0.23 ± 0.01	0.21 ± 0.04	0.23 ± 0.03	0.19 ± 0.03	0.227
C18:1n7	0.92 ± 0.04	1.00 ± 0.05	1.01 ± 0.10	1.01 ± 0.05	0.180
C18:1n9	38.00 ± 0.29 ^a^	38.84 ± 0.45 ^b^	38.80 ± 0.22 ^b^	38.97 ± 0.49 ^b^	0.014
C20:1	0.48 ± 0.03 ^ab^	0.52 ± 0.03 ^b^	0.46 ± 0.04 ^a^	0.42 ± 0.04 ^a^	0.010
C18:2n6	10.30 ± 0.21 ^A^	10.06 ± 0.10 ^A^	10.70 ± 0.23 ^B^	10.80 ± 0.18 ^B^	<0.001
C18:3n3	0.13 ± 0.01	0.13 ± 0.01	0.13 ± 0.01	0.12 ± 0.01	0.262
C18:3n6	0.27 ± 0.02	0.32 ± 0.02	0.30 ± 0.03	0.30 ± 0.03	0.071
C20:3	0.14 ± 0.02	0.15 ± 0.02	0.14 ± 0.01	0.16 ± 0.02	0.355
C20:4	2.60 ± 0.09	2.58 ± 0.13	2.63 ± 0.11	2.46 ± 0.07	0.168
MUFA ^1^	45.39 ± 0.29 ^A^	46.65 ± 0.46 ^B^	46.40 ± 0.18 ^B^	46.53 ± 0.50 ^B^	0.002
PUFA ^2^	13.43 ± 0.27 ^ab^	13.24 ± 0.19 ^a^	13.90 ± 0.35 ^c^	13.85 ± 0.26 ^bc^	0.013
SFA ^3^	41.18 ± 0.37 ^B^	40.12 ± 0.30 ^A^	39.70 ± 0.19 ^A^	39.62 ± 0.38 ^A^	<0.001
UFA:SFA ^4^	1.43 ± 0.02 ^A^	1.49 ± 0.02 ^B^	1.52 ± 0.01 ^BC^	1.52 ± 0.02 ^C^	<0.001

Note: Data are expressed as means ± standard deviation, *n* = 4. ^1^ MUFA = C16:1 + C17:1 + C18:1n7 + C18:1n9 + C20:1; ^2^ PUFA = C18:2n6 + C18:3n3 + C18:3n6 + C20:3 + C20:4; ^3^ SFA = C10:0 + C12:0 + C14:0 + C16:0 + C17:0 + C18:0 + C20:0; ^4^ UFA = MUFA + PUFA. CON = basal diet; FNOP = fermented navel orange pulp; MUFA = monounsaturated fatty acid; PUFA = polyunsaturated fatty acid; SFA = saturated fatty acid. ^a,b,c^ or ^A,B,C^ Within a row, values with different superscripts differ significantly at *p* < 0.05 and *p* < 0.01, respectively.

**Table 9:** Effects of fermented navel orange pulp on the serum biochemical indexes of finishing Tibetan pigs.

Item	CON	5% FNOP	10% FNOP	15% FNOP	*p*-Value
TP, ug/uL	73.46 ± 8.18 ^A^	89.17 ± 9.36 ^B^	82.33 ± 9.02 ^B^	84.42 ± 9.59 ^B^	0.001
BUN, mmol/L	7.59 ± 1.48	7.30 ± 1.71	7.59 ± 1.03	7.80 ± 1.35	0.865
UA, mmol/L	23.72 ± 12.47 ^A^	42.24 ± 16.13 ^B^	43.11 ± 10.35 ^B^	49.20 ± 25.73 ^B^	0.007
TG, mmol/L	0.59 ± 0.24 ^a^	0.69 ± 0.29 ^ab^	0.78 ± 0.13 ^ab^	0.92 ± 0.37 ^b^	0.036
GH, ug/L	11.85 ± 1.62 ^C^	10.84 ± 2.69 ^BC^	9.66 ± 1.58 ^B^	7.48 ± 1.00 ^A^	0.002
IGF-1, μg/L	9.33 ± 1.43 ^b^	9.69 ± 1.35 ^b^	9.06 ± 1.69 ^ab^	7.82 ± 1.19 ^a^	0.040

Note: Data are expressed as means ± standard deviation, *n* = 12. CON = basal diet; FNOP = fermented navel orange pulp; TP = total protein; BUN = blood urea nitrogen; UA = uric acid; TG = triglyceride; GH = growth hormone; IGF-1 = insulin-like growth factor-1. ^a,b^ or ^A,B,C^ Within a row, values with different superscripts differ significantly at *p* < 0.05 and *p* < 0.01, respectively.

## Data Availability

The original contributions presented in this study are included in the article; further inquiries can be directed to the corresponding authors.
